# Dynamic Reweighting of Auditory Modulation Filters

**DOI:** 10.1371/journal.pcbi.1005019

**Published:** 2016-07-11

**Authors:** Eva R. M. Joosten, Shihab A. Shamma, Christian Lorenzi, Peter Neri

**Affiliations:** 1 Laboratoire Psychologie de la Perception (CNRS UMR 8242) and Université Paris Descartes, Sorbonne Paris Cité, Paris, France; 2 Laboratoire des Systèmes Perceptifs (CNRS UMR 8248) and Département d’études cognitives, Ecole Normale Supérieure, PSL Research University, Paris, France; 3 Department of Electrical and Computer Engineering, Institute for Systems Research, University of Maryland, College Park, Maryland, United States of America; Technische Universitat Chemnitz, GERMANY

## Abstract

Sound waveforms convey information largely via amplitude modulations (AM). A large body of experimental evidence has provided support for a modulation (bandpass) filterbank. Details of this model have varied over time partly reflecting different experimental conditions and diverse datasets from distinct task strategies, contributing uncertainty to the bandwidth measurements and leaving important issues unresolved. We adopt here a solely data-driven measurement approach in which we first demonstrate how different models can be subsumed within a common ‘cascade’ framework, and then proceed to characterize the cascade via system identification analysis using a single stimulus/task specification and hence stable task rules largely unconstrained by any model or parameters. Observers were required to detect a brief change in level superimposed onto random level changes that served as AM noise; the relationship between trial-by-trial noisy fluctuations and corresponding human responses enables targeted identification of distinct cascade elements. The resulting measurements exhibit a dynamic complex picture in which human perception of auditory modulations appears adaptive in nature, evolving from an initial lowpass to bandpass modes (with broad tuning, Q∼1) following repeated stimulus exposure.

## Author Summary

Amplitude modulations are considered the key carriers of intelligible information in auditory signals, and consequently it is of significant interest to discover how they are neurally analyzed and perceptually encoded. A dominant model has emerged from extensive experimental and theoretical studies of this phenomenon. This model posits that amplitude modulations are parsed into channels of different temporal rates via a bank of bandpass filters. Using exclusively data driven approaches with minimal assumptions about the structure of the model, the picture that emerges is of an adaptive process. Initially, human listeners in these tasks perceive modulations as if through a lowpass filter with very low cutoff frequency, which gradually evolves to become a broadly tuned bandpass process at higher modulation frequencies, reflecting the modulations of the target stimuli. This surprising dynamic characteristic emphasizes the plastic nature of modulation analysis in sensory perception.

## Introduction

Natural sounds carry salient amplitude modulations (AM) essential for successful interpretation of auditory landscapes and robust source identification [1–5]. The human auditory system is exquisitely sensitive to relatively slow AM cues, prompting extensive investigation of this ability over several decades [6, 7]. Recent success in modeling recognition of environmental sound textures [8], speech [9–11] and music material [12–14] has now prompted the need to consolidate the exact nature of modulation filtering [15–18].

Early measurements of sensitivity across AM rates seemed consistent with low-pass characteristics (running average over time) as envisaged by the ‘linear envelope detector’ (LED) model [6, 19, 20]. However, this class of experiments often posed interpretational challenges. For example, under some stimulus and experimental conditions only parts of the dataset conformed to low-pass characteristics [6, 15, 21–23] but not others [16, 24, 25]. Plausible accounts of these apparent inconsistencies involved shifts in task strategies and/or decision statistics [15, 21], whereby listeners would rely on different cues under conditions associated with different portions of the dataset and/or subtle stimulus alterations [26–28]. Strategy shifts of this kind are relatively common [29] (e.g. off-frequency listening [30]). Therefore, our first goal was to assess the extent to which *endogenous* adaptive strategies can influence the measurements of the modulation filter when no stimulus and/or task-related factors are concomitantly modified. Specifically, we sought to adopt a single stable measurement approach throughout (the *reverse correlation* method), reasoning that systematic changes in the subjects’ detection strategies would be unveiled as a dynamic evolution of the filter estimates during the experimental session [31–33].

The shortcomings of the low-pass AM filter model have since spurred development of alternative models which augmented it with, for example, an autocorrelation stage [34], or the current dominant framework of the modulation filterbank (MFB) [16, 35]. This latter model consists of a low-pass filter (up to 2.5 Hz) and a bank of band-pass filters spanning the ∼5–100 Hz range. Thanks to its increased sophistication and flexibility, the MFB is able to account for a wide range of psychophysical [16, 24, 36–39] and physiological [35, 40–42] results. Nevertheless, there remain several important details of these hypothesized filters that have not been adequately constrained by available data. For instance, filter tuning (Q-value) has often been assumed to measure ∼2 [35], yet other studies have indicated a significantly lower value (<1) [25]. Furthermore, another study set out to test the ability of the modulation filter bank model to account for dependence of AM envelope detection on the nature of the carrier. It reported that the model significantly underestimated the detrimental effects of carrier periodicity on modulation detection, a finding that has cast a shadow of uncertainty on certain aspects of the filterbank model [34]. Another unexplained finding concerns the lack of persistent low-level adaptive effects in these postulated filters [18], suggesting that they are more dynamic and likely more susceptible to cognitive control (e.g. by the details of the measurement task [43]). With specific relation to psychoacoustical literature using decision weights [44] (a technique related to the reverse correlation approach used here), the bandpass signatures expected of the MFB model are not directly evident through previous filter estimates [45–48] (we return to this issue in Discussion). Consequently, the second goal of the present study is to measure these bandpass filters in an unconstrained manner to allow for data-driven conclusions that are largely independent of model specifics. To do so, we relied on a combination of powerful system identification tools and AM-tailored stimulus perturbations that enabled us to describe a more dynamic picture of the underlying process encompassing both LED and MFB modes of operation.

Our findings largely confirm the three critical questions we sought to address: (1) the measured bandpass channels undergo characteristic changes to reflect listeners’ strategy shifts from using a combination of loudness and spectral-profile cues (both existing in the presented stimuli) in the early part of the experiment, to relying primarily on the spectral-profile cue during later phases; (2) the Q-values associated with these channels are ∼1, although this characteristic is itself subject to recalibration as assessed by our measurement task and analysis; and (3) the modulation filter bank (augmented by a low-pass characteristic with a low cut-off [35]) remains the most parsimonious model for auditory modulation processing.

## Materials and Methods

### Ethics statement

Ethics approval was obtained from the College Ethics Review Board (CERB) at Aberdeen University (http://www.abdn.ac.uk/clsm/working-here/cerb.php). All listeners gave written informed consent.

### Stimulus and task

**Fig 1:**
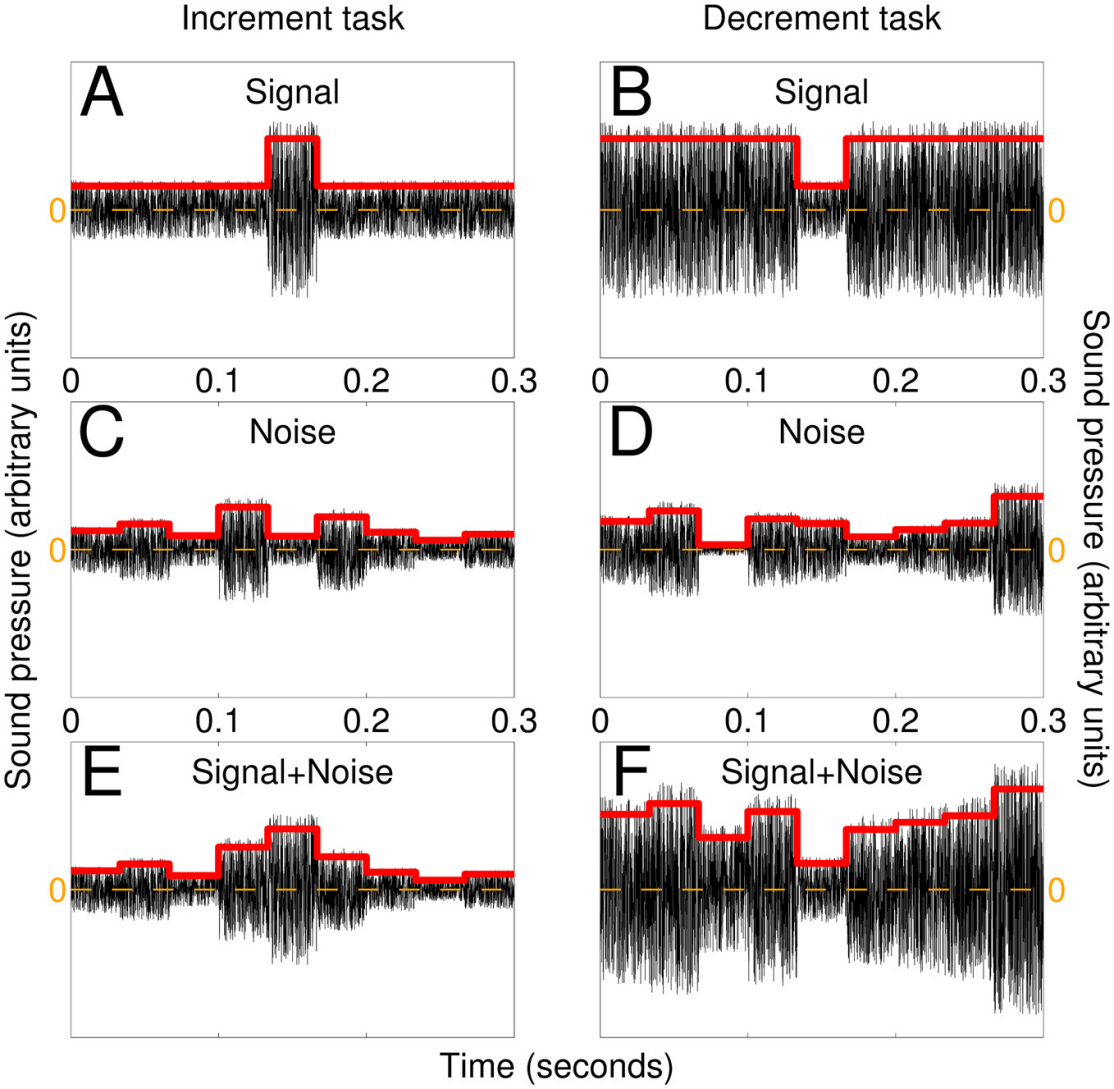
AM modulations as targets and interferers. The signal to be detected consisted of a centered 33-ms square-pulse change in AM envelope that was either larger (A) or smaller (B) than baseline modulation depending on whether listeners were engaged in the increment or decrement task respectively. (C,D) Noise was applied in the form of Gaussian modulations around baseline value every 33-ms to generate signal+noise stimuli (E-F). Black traces show sound waves, red traces show amplitude modulations. See Materials and Methods for details.

The auditory stimuli (delivered binaurally via Sennheiser HD202 headphones) were specifically designed to encode task-relevant AM modulations into the envelope of the acoustic signal in a manner suitable for experimental characterization using psychophysical reverse correlation [49], and their parameters were specified to lie in the perceptually salient range for AM processing (<30 Hz). The 300-ms carrier was a segment of white noise (5 kHz bandwidth, 10 kHz sampling rate) that was generated once at the beginning of each block and identically replicated throughout the entire block; on any given block, no variability/perturbation was therefore introduced by the fine temporal structure of the stimulus (see further below and S6 Fig for additional analyses demonstrating that carrier-induced AM deviations had no impact on our results). Stimulus perturbation was instead applied to the overall amplitude of the carrier in a stepwise fashion: the stimulus waveform was subdivided into 9 temporal segments (each lasting ∼30-ms) and the amplitude of each segment was controlled independently [46]. In the absence of an applied increment/decrement (signal), a given segment was assigned a fixed baseline level (indicated by leading and trailing ends of red line in Fig 1A) of ∼62 dB SPL. The ‘increment’ signal was generated by increasing the amplitude of the central segment within the stimulus waveform (peak of red line in Fig 1A) to ∼68 dB SPL (we use ∼ because the exact values were tailored to each listener to target individual threshold performance and therefore differed across listeners); the temporal scale of this modulation was chosen to match ecologically relevant AM cues [1, 50], and its AM pulse-like specification was chosen to ease application of analytical/theoretical results dependent on signal shape [51]. The target signal was then added to a noise waveform generated by applying a random Gaussian-distributed increment/decrement to the overall amplitude of each segment, independently for different segments (Fig 1C); the resulting signal+noise trace (Fig 1E) was presented together with a noise-only trace on every trial, and listeners were asked to detect the former in a two-interval-forced-choice (2IFC) protocol. The jitter introduced by the noisy modulation was approximately ±2.2 dB standard deviation around baseline level. In a separate series of experiments we asked the same listeners to detect a signal defined by a decrement (rather than an increment) in the amplitude of the central segment (Fig 1B) from a baseline level of ∼68 dB to ∼62 dB. Some previous studies on AM minimized the role of loudness cues by roving the absolute levels of individual stimuli [44, 52]; we deliberately avoided this manipulation because it amplifies the role of gain control [53], a nonlinear effect that may have compromised interpretation of the nonlinear kernels [51, 54]: roving stimuli span a wide level range for the purpose of rendering overall absolute level ineffective as a cue for performing discrimination, however they also require listeners to *factor out* overall level via gain control (or analogous mechanisms) to compute relative filter outputs; this dynamic nonlinear operation is difficult to incorporate into the cascade models that form the theoretical underpinnings of the present study [51, 58], potentially complicating kernel analysis beyond the level of interpretability afforded by analyses like those favoured here that do not involve parameter fitting. Following their response (via button press), auditory feedback (correct/incorrect) was provided immediately; the next trial automatically initiated after a 1-second delay. At the end of each block, listeners received an auditory summary (via an automated system based on segments of human voice) detailing the total number of collected trials and the percentage of correct responses on the last block as well as averaged across all blocks.

### Targeting optimal performance regimes

We tested 10 listeners, all naive except author EJ (indicated by square symbol in all Figures), with ages 28±9 years (mean±SD across listeners). Listeners were initially familiarized with the task during preliminary training sessions consisting of ∼20 trials without noise. They were presented with noiseless versions of both signal+noise and noise-only stimuli, preceded by explicit verbal indication of whether they would hear the former or the latter. They were then asked to indicate the signal+noise interval and provided with trial-by-trial feedback (correct/incorrect). After this preliminary phase, we adjusted noise levels individually to target optimal threshold regimes for the deployment of reverse correlation [55] (S1 Fig). Percent correct was 74%±6% (increment detection, 5.5k±2.8k trials per listener) and 76%±7% (decrement detection, 3.6k±3.3k trials per listener). We successfully minimized response bias across listeners (S1B Fig); this is particularly important when attempting nonlinear system identification to avoid bias-induced modulations within nonlinear kernels: in the presence of bias, 2^*nd*^ order kernels (see below for detailed description/definition) may contain modulations that do not reflect the structure of the perceptual machinery preceding the binary behavioural decision, but are instead produced by the nonlinear nature of the conversion from decision variable to binary output which, in general, is not relevant for characterizing the perceptual machinery itself as it is protocol-specific [51] (i.e. it depends on the arbitrary way in which listeners are asked to express their percept, whether via binary choice (‘yes’/‘no’, ‘present’/‘absent’) or rating scale, for example). Internal noise was within the expected range for human psychophysics [56] (S1C Fig), indicating that listeners adopted a robust task strategy, as also evidenced by the relatively high absolute efficiency (within the normal range for detection [57] and much higher than observed for other auditory tasks [58], see S1D Fig). All aspects of performance metric analysis indicate that 1) listeners performed the above-detailed tasks in a stable and efficient manner, with no discernible difference in overall performance metrics between increment and decrement experiments (data points fall around diagonal unity line in S1 Fig); 2) our protocols successfully established optimal conditions for the application of behavioural reverse correlation [49, 51, 56].

### LED/MFB models recast as LNL cascades

**Fig 2:**
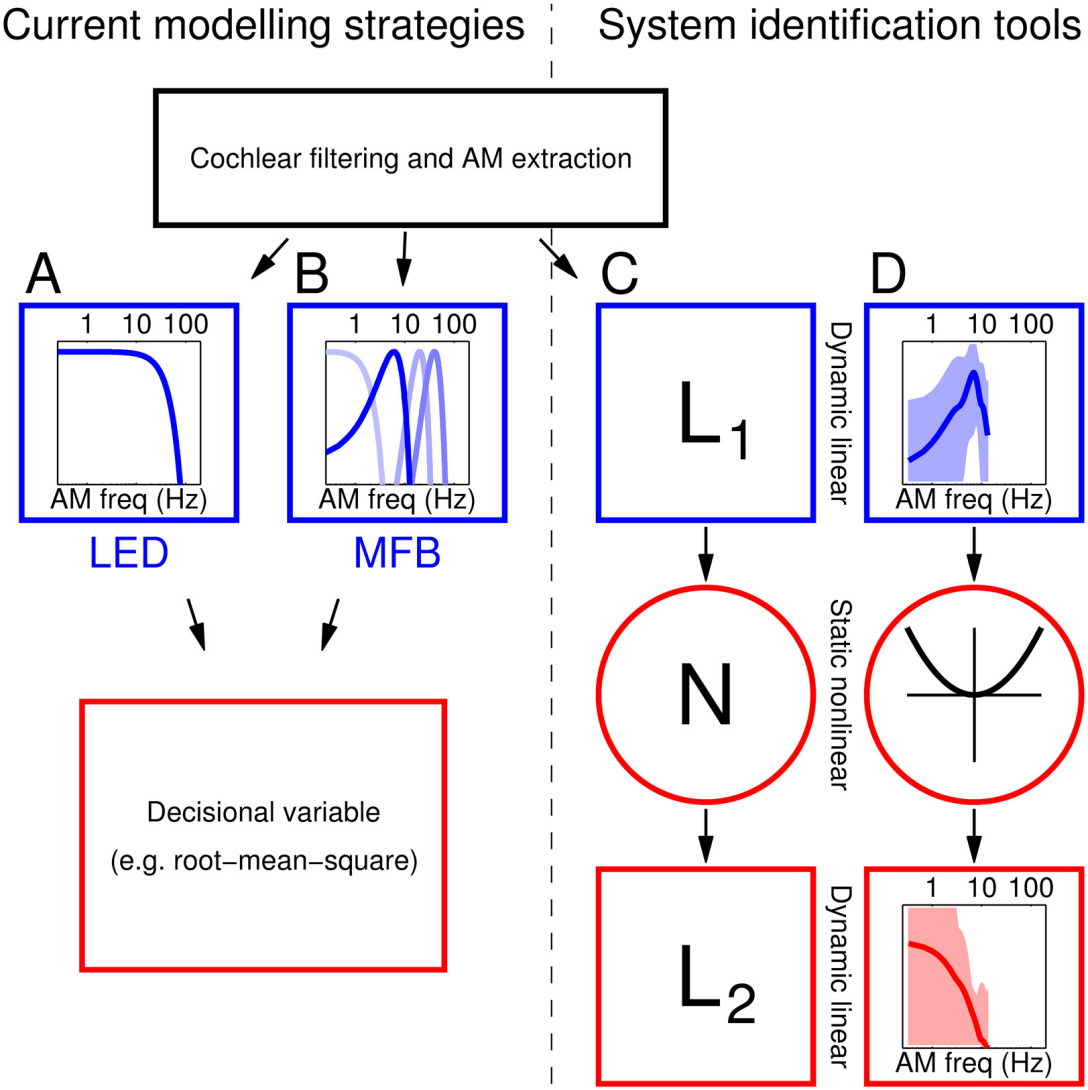
Models of AM processing in the auditory system. All such models consist of a cochlear filtering and AM envelope extraction stage (top), followed by one of two competing formulations: (A) a linear lowpass filter that smooths the AM envelope, better known as the linear envelope detector (LED) model; or (B) a modulation frequency bank (MFB) model that bandpass filters the AM rates into different channels. Both models feed into a decisional stage (red). (C) They can be reformulated as a cascade of linear-nonlinear-linear (**L**_1_**NL**_2_) stages, with the additional combined nonlinear-linear (**NL**_2_) operation accommodating different decisional rules, e.g. squaring followed by sum in the case of energy-based rules. When formulated as such, the two standard models in (A-B) are primarily distinguished by the nature of their **L**_1_ modules (blue): lowpass in one and bank of bandpass filters in the other. (D) Our cumulative empirical estimates of **L**_1_ and **L**_2_ support MFB characteristics (blue trace in D is compatible with blue traces in B, not A).

**Fig 3:**
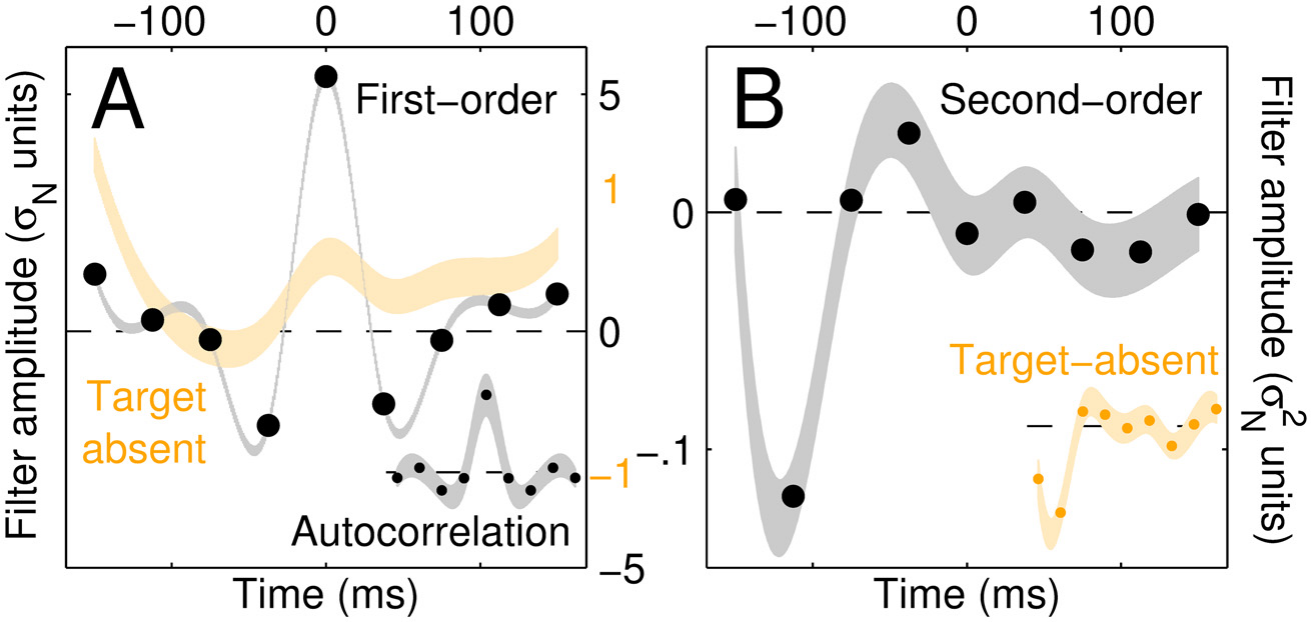
Measured kernels and associated system identification estimates of L_1_. Target-present/target-absent (black/orange) first-order kernels for increment detection are plotted in A (shaded regions show ±1 SEM). B plots first row from second-order kernel (or equivalently column, see S2C–S2E Fig); inset (orange) shows same from target-absent data. The black trace in A is an estimate of the autocorrelation of **L**_1_ [51], while the trace in B is an (independent) estimate of **L**_1_ itself [59, 64]. To confirm this, we plot the autocorrelation of B in the inset of A: it matches the black trace shown in the main panel of A (see Materials and Methods for details).

The modulation filtering models are referred to as LED/MFB models. They are normally implemented as illustrated in Fig 2. For the purpose of examining how these models relate to our protocols, we treat the incoming stimulus as defined in AM space (i.e. as a 9-element vector where individual entries indicate the amplitude of each segment) because this is the stimulus subspace within which input noise was applied (see Fig 1); in other words, cochlear filtering (top in Fig 2) is reduced by our stimulus generation protocols, because noise is applied not to the fine structure of the stimulus waveform but to its AM profile. The LED involves application of a low-pass filter [6] to the (modulation) envelope, while the MFB applies primarily band-pass filtering [16]. This front-end stage is illustrated in Fig 2A and 2B for LED and MFB respectively. With reference to the 9-element input vector where each entry refers to a different time point, this filtering stage consists of convolution between this input vector and a temporal impulse response **L**_1_. We represent the filter frequency characteristics in Fig 2 (blue) because they are easier to interpret as lowpass/bandpass, but our **L**_1_ estimates are initially recovered as temporal impulse responses (Fig 3B) because they are obtained via direct reverse-correlation of the input stimulus (defined across time). The output from the **L**_1_ layer (which is itself a function of time) is then passed onto a decision stage that generates a psychophysical response (red rectangle in Fig 2). The details of how this stage operates are still unclear [15, 21]. We can describe the sequence of operations carried out by LED/MFB models using the same general cascade, as illustrated in Fig 2C. In this formulation, the LED/MFB filters correspond to different characteristics for the first filtering stage **L**_1_. Subsequent decision stages are approximated by a combined nonlinear-linear operation (red outlines in Fig 2C). For example, if read-out involves energy extraction from the temporal output returned by **L**_1_, **N** corresponds to squaring and **L**_2_ to sum over time. Similar approximations can be adopted for root-mean-square and MAX rules [51, 58]. The two filters **L**_1_ and **L**_2_ are referred to as the *cascade filters*. Our goal is to estimate their structure via the *psychophysical kernels* we can measure from data (see below); this is achieved by exploiting a set of analytical tools that establish important connections between cascade filters and psychophysical kernels [59].

### Derivation of psychophysical kernels

Psychophysical kernels are used as compact descriptors of stimulus properties that impact listeners’ decisions in simple detection/discrimination tasks [51, 60]. The most effective approach to an intuitive understanding of the 1^st^ order kernel is to think of the underlying perceptual process as a matched template that assigns a set of weights to different elements of the stimulus, sums across all elements, and finally converts this weighted sum into a binary decision of the kind ‘I saw the target’ or ‘I did not see it’ [49, 61]. For this model, the psychophysical 1^st^ order kernel (computed as described below) is an image of the template [49, 60]: it details the perceptual impact associated with different portions of the auditory waveform. It also does not matter whether the kernel is computed from noise modulations associated with the target or not: in both cases, it will reflect the template associated with the model outlined above, if this model provides an adequate account of the perceptual process [49, 60]. There are many conditions, however, when the 1^st^ order kernel does not retain the intuitively transparent interpretation proposed above, e.g. in the presence of a nonlinear transformation between the stimulus and the response such as a dependence on the power or correlational structure of the stimulus [62, 63]. In order to extract useful information about the underlying process, it then becomes necessary to study higher-order descriptors such as the 2^nd^ or 3^rd^ order kernels [64], requiring more data and elaborate models. For example, the 2^nd^ order kernel is useful if we suspect that the perceptual process assigns a set of weights to all possible pairwise *interactions* between different elements of the stimulus, e.g. between the amplitudes of the first and the second segments of the auditory waveform, or between the amplitudes of the first and third segments, and so on. These interaction terms would provide additional information about the stimulus properties that affect listeners’ choices beyond the description afforded by the 1^st^ order kernels [51, 59, 64]. To compute 1^st^ and 2^nd^ order kernels, we denote the AM noise modulation applied on the target-present (*q* = 1) or target-absent (*q* = 0) interval of a trial to which listeners responded correctly (*r* = 1) or incorrectly (*r* = 0) by the 9-element vector **n**^[*q*,*r*]^. The first-order target-present psychophysical kernels (i.e. those obtained *only* from noise trials containing the target) were computed as $p_{1}^{\left[1\right]}=\text{avg}\left(n^{\left[1,1\right]}\right)-\text{avg}\left(n^{\left[1,0\right]}\right)$ where avg(.) is used to indicate average across trials of the specified type [49]; the target-absent kernels were $p_{1}^{\left[0\right]}=\text{avg}\left(n^{\left[0,0\right]}\right)-\text{avg}\left(n^{\left[0,1\right]}\right)$. The second-order psychophysical kernels were similarly computed as **p**_2_ = cov(**n**^[1,1]^) + cov(**n**^[0,0]^) − cov(**n**^[1,0]^) − cov(**n**^[0,1]^) where cov(.) indicates covariance across trials. Please see [51, 60] for further details of these methods.

### L_1_/L_2_ estimates via cascade characterization

Cascade filters and psychophysical kernels are different classes of objects. Cascade filters are filtering components of a hypothesized cascade model; for the **L**_1_**NL**_2_ cascade described previously, they cannot be estimated directly from data via simple rules. They can, however, be estimated *indirectly* via the psychophysical kernels. Psychophysical kernels are data descriptors computed directly from the raw data using simple rules (see above); in this sense, they are not dissimilar from simply computing a summary statistic (e.g. mean or median) from a dataset. Their estimation is robust and does not depend on any assumed underlying model. If a model is assumed, the kernels can then be used to characterize specific components of the model. For example, the **LN** model is widely adopted for this type of application [49]; in its psychophysical variant, this model reduces to template matching for the **L** stage [51], i.e. inner product between the input stimulus and the **L** template [65–67]. Under this model, the first-order psychophysical kernel returns a scaled image of the template **L** [60], allowing for direct transparent estimation of the linear filtering stage.

For the purpose of our study the **LN** model is inapplicable as it predicts [49, 51, 58, 68, 69] that perceptual kernels derived from noise modulations associated with signal+noise stimuli (‘target-present’) must match those derived from noise-only stimuli (‘target-absent’). This property is a consequence of the linear nature of the **L** stage, combined with the classic reverse-correlation result that the static nonlinear **N** stage is bypassed by the kernel estimation procedure [60, 70, 71]. The response of **L** to signal+noise is simply the sum of its response to signal plus its response to noise, effectively decoupling the noise-driven response from that associated with the signal. The filter perturbation associated with the noise element is therefore statistically analogous between signal+noise and noise-only stimuli, leading to equivalent kernel estimates [68, 69]. This prediction is not born out by the results in S2A and S2B Fig which show that target-absent and target-present first-order kernels respectively are markedly different (compare not only the shape of the traces, but also the scaling of ordinate between S2A and S2B Fig; see also [72] and S7A Fig). This finding is consistent with the expectation that the underlying mechanism would more likely conform to cascade models including additional filters, e.g. the **L**_1_**NL**_2_ cascade in Fig 2C (see more below); because this cascade does not belong to the **LN** family of models, it does not predict that target-present and target-absent 1^st^ order kernels should match but rather that (in general) they should differ [51], as we observe.

Under the **L**_1_**NL**_2_ cascade model, the connection between filter components (**L**_1_/**L**_2_) and psychophysical kernels ($p_{1}^{\left[1\right]}$/$p_{1}^{\left[0\right]}$/**p**_2_) is provided by the following three theoretical results (#1-2 pertaining to **L**_1_, #3 pertaining primarily to **L**_2_). Result #1: target-present first-order kernels (Fig 3A) return an approximate image of **L**_1_ autocorrelation [51]; we can exploit this result to study the characteristics of **L**_1_, with the cautionary note that the relationship between $p_{1}^{\left[1\right]}$ and **L**_1_ involves other terms besides **L**_1_ autocorrelation and that the relative contribution of these terms depends on stimulus SNR [51]. Result #2: **L**_1_ can also be estimated from *second-order* kernels by exploiting the established result that the first row (or column) of the second-order kernel (black and red rectangles in S2C and S2D Fig, replotted as traces in Fig 3B) returns an approximate image of **L**_1_ [51, 59, 64]. This is the approach typically adopted for solving **L**_1_**NL**_2_ cascades [59, 64]; we conform to this practice by relying primarily on these **L**_1_ estimates here. We can cross-check the consistency of **L**_1_ estimates returned by these two approaches: if we take the autocorrelation of the trace in Fig 3B and plot it in the inset to Fig 3A, it should resemble the trace in Fig 3A. This prediction is well realized by data (see also S2B and S2E Fig), thus lending further support to the applicability of the associated analytical tools to the present context.

**Fig 4:**
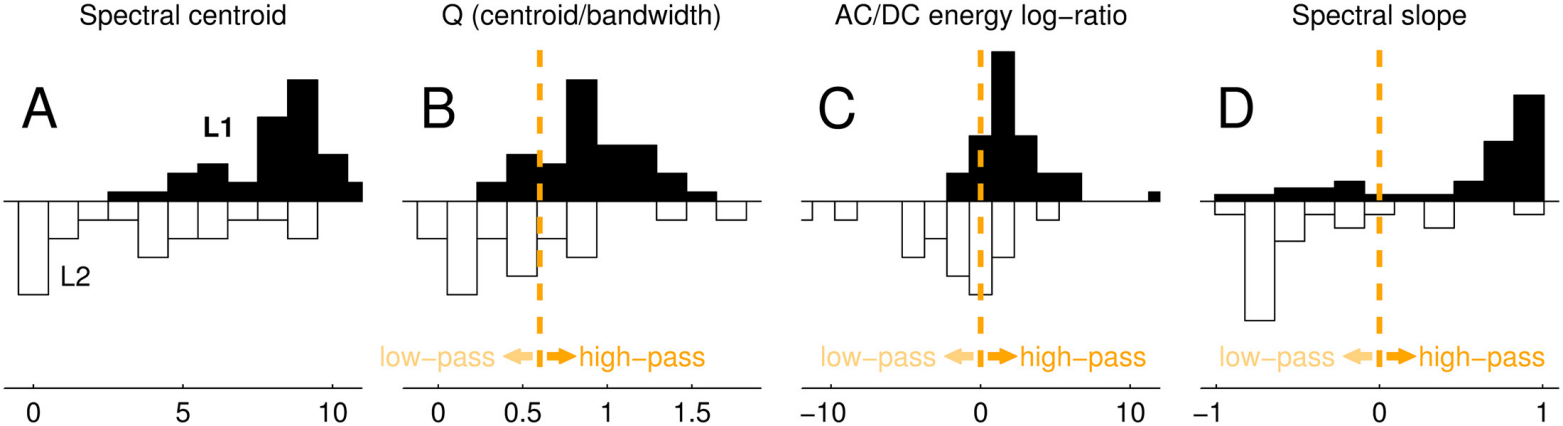
The band-pass character of L_1_. The band-pass characteristics of the **L**_1_ filter, contrasted with the low-pass **L**_2_ read-out filter, are demonstrated by the distribution of four filter parameters (centroid in A, Q in B, AC/DC energy log-ratio in C, spectral slope in D) applied to all **L**_1_/**L**_2_ estimates. Parameters are gathered from across all experiments (increment/decrement detection) and listeners. Vertical dashed lines in panels (B,C,D) mark transition from low-pass to high-pass for each given parameter (see Materials and Methods for details).

**Fig 5:**
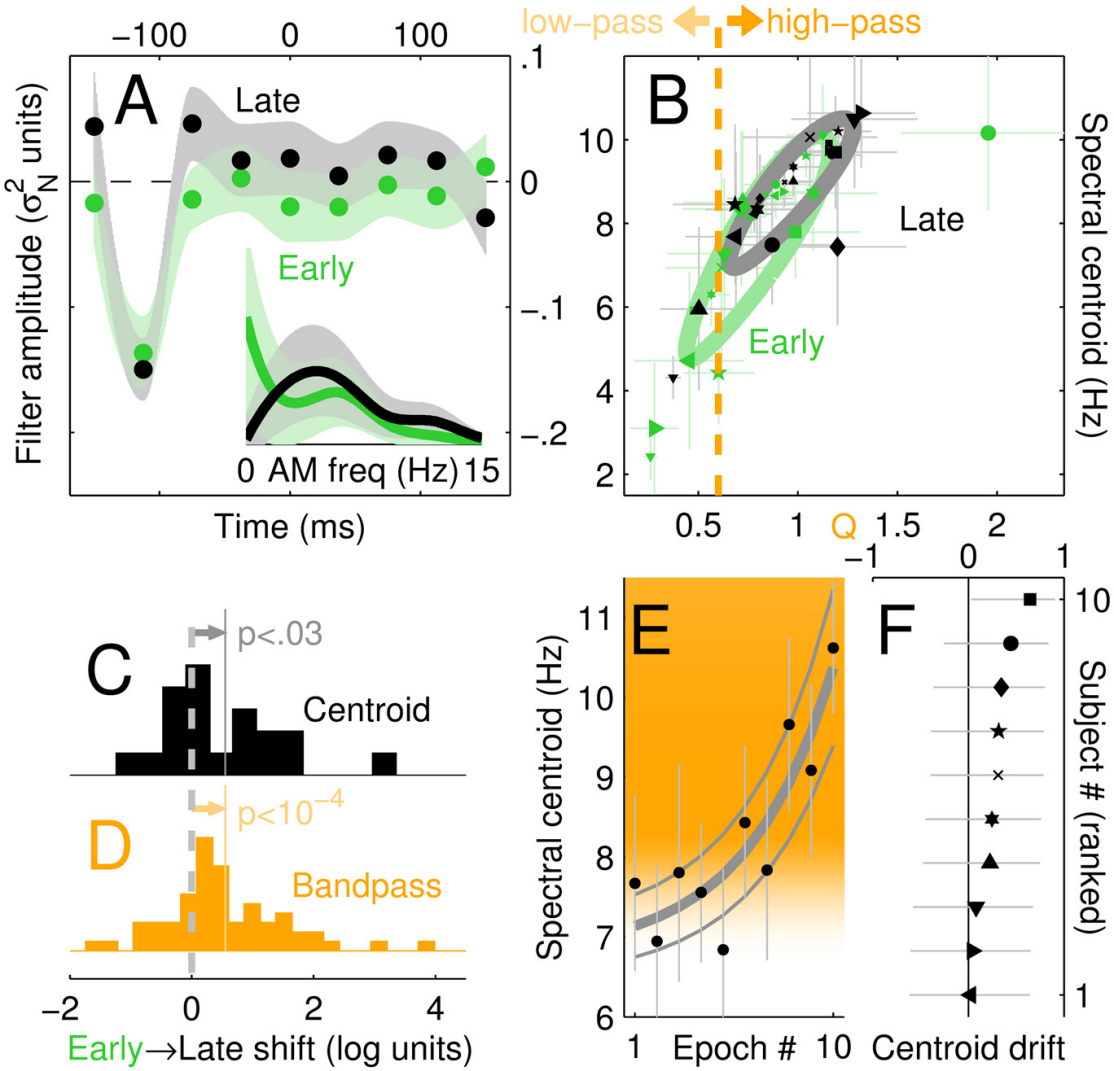
Front-end filter (L_1_) shifts from low-pass to band-pass characteristics with exposure. (A) **L**_1_ estimates as in Fig 3B, separately for ‘early’ (green) and ‘late’ (black) phases of the data collection epochs (see Materials and Methods). Inset curves show the corresponding low-pass (early) and band-pass (late) power spectra of the two **L**_1_ estimates. (B) Quantifying the shift from low-pass to band-pass shapes of the **L**_1_ filter. The spectral centroid (y axis) is plotted against the Q estimates using the same symbols as in Fig 4. Ovals are aligned with best-fit line, with radii matched to SD’s for data projected parallel and orthogonal to line. (C) Distribution of late/early log-ratios for centroid values. (D) Distribution of late/early comparisons for all three band-pass metrics (Q, AC/DC energy log-ratio, spectral slope). (E) Gradual change in the spectral centroids in 10 successive epochs during the experiments (rather than just early/late); orange shading reflects task-relevant stimulus modulations across centroid values in the bandpass region (see Materials and Methods and S5I Fig for a full plot of these modulations spanning the low frequency region). Solid line shows semi-log linear fit (thin lines plot boundaries ±1 SEM around fit parameters). Associated correlation coefficient (centroid drift) is plotted separately for each subject in (F). Error bars/margins plot ±1 SEM in (A-B, E); ±95% confidence intervals in (F).

We can also estimate **L**_2_ by relying on the additional result (#3) that target-absent first-order psychophysical kernels (S2A Fig) return the cross-correlation between **L**_1_ and **L**_2_, as detailed in [51, 73]. We can then deconvolve the **L**_1_ estimates (obtained as described above) out of target-absent first-order kernels to obtain estimates for **L**_2_. The additional deconvolution step involved in deriving **L**_2_ partly justifies the noisiness associated with the aggregate estimate in Fig 2D (shading shows ±1 SEM; this is compounded by computing the power spectrum from time-based filter estimates before combining them across listeners, who naturally displayed a significant degree of individual variability). Notice that all our conclusions are based on quantitative analysis of individual listener data (Figs 4, 5B–5D and 5F); aggregate estimates are shown for visualization purposes only.

In practice, **L**_1_/**L**_2_ estimates will be distorted images of the kernels associated with theoretical accounts of **L**_1_**NL**_2_ cascades due to analytical approximations [51, 73] and the highly nonlinear properties of AM extraction [16], but the qualitative nature of their filtering characteristics (whether low-, high- or band-pass) is preserved in the presence of these distortions (as we have verified via Monte Carlo simulations of full-scale models explicitly encompassing all stages from cochlear filtering to binary choice; see S5 Fig for a diagrammatic representation of one such simulation).

### Scalar metrics for quantitative evaluation of L_1_/L_2_ estimates

We computed the power spectrum **w** of each **L**_1_ and **L**_2_ estimate and gauged its band-pass characteristics using four parameter-free metrics; because target-present kernels (Fig 3A) approximate **L**_1_ autocorrelation, the corresponding power spectrum was obtained via Fourier transform [74]. The spectral centroid (Fig 4A) was $f•\hat{w}$ where **f** is the vector of sampled spatial frequencies and $\hat{w}$ was obtained by normalizing **w** to sum 1. The ratio between spectral centroid and SD of $\hat{w}$ provides a surrogate index of band-pass characteristics for bell-shaped $\hat{w}$; the transition value from low-pass to high-pass (marked by vertical dashed line in Fig 4B) corresponds to a uniform spectrum, for which the centroid/SD ratio is $\sqrt{8}/2$ given the sampling rate used here. To establish a link with existing literature, in Fig 4B we plot this quantity in units of Q, the mean/width ratio for a rectangular shape; the conversion is obtained by approximating an assumed Gaussian shape with a rectangle of equivalent full-width at half-height (FWHH) [7], for which the conversion factor is $\text{FWHH}=\text{SD}\times 2\sqrt{2\text{log}(2)}$. AC/DC energy log-ratio (Fig 4C) was log[**w**(**f** > 0)/**w**(0)] where **w**(0) is power at frequency 0 (DC) and **w**(**f** > 0) is all remaining power in the spectrum. Spectral slope (Fig 4D) was the correlation coefficient of **w** across **f**. The composite band-pass index in Fig 5D consisted of paired comparisons between ‘early’ and ‘late’ estimates of Q, AC/DC energy log-ratio and spectral slope; because Q is always positive the comparison involved log-ratios, while AC/DC energy log-ratios and spectral slopes (can be negative) were compared via subtraction.

### Epoch definition and related analysis

Increment and decrement experiments were often run in alternate fashion across sessions (although they were never mixed within the same session/day); for exposure-related analyses (Fig 5), they were combined following sign inversion for noise modulations from decrement experiments to align them with the increment data. We restricted our analysis to the smallest number of trials collected by any listener (3.5k) to make the analysis comparable across listeners (35k trials contributed to Fig 5). We split this initial period of data collection into ‘early’ and ‘late’ epochs by assigning the first 1750 trials to the former and the second 1750 trials to the latter. For the 10-epoch analysis (Fig 5E and 5F) we split the same period into 10 epochs of 350 trials each. Centroid drift (x axis in Fig 5F) is the correlation coefficient of centroid versus logarithm of epoch number. We logged epoch number before computing correlation because the semilogarithmic representation corresponded to an excellent linear fit of the aggregate data in Fig 5E (see solid gray lines).

### Coupling between noise DC fluctuations and trial-by-trial responses

We averaged noise amplitude (as specified by the 9-element vector **n** detailed above) and used it as proxy for the noise-induced DC perturbation of each stimulus. We then took the difference in noise DC content between the two stimuli presented on each trial, and computed the biserial correlation coefficient between this differential DC content and the binary response returned by listeners (S4B Fig). We applied the above calculation only to the noise component of each stimulus (without target signal) because, when the signal is included, DC content is almost invariably greater for the stimulus containing the target signal; given that listeners performed above chance (i.e. their response was correlated with target presence), it is trivial that we should find a correlation (which we do find in all instances) between differential target-driven DC content and behavioural response. We therefore focused on the behavioural component of the response that was specifically driven by trial-to-trial random fluctuations of DC content (i.e. those induced by the noise), rather than the expected correlation with target presence.

### Stimulus bandwidth characterization and correction for carrier-induced AM deviations

We estimated stimulus task-relevant modulation for different centroid values by extracting the AM content of signal+noise and noise-only stimulus waveforms via a bank of 1-octave AM filters centred at 2, 4, 8, 16 and 32 Hz, plus a lowpass filter with a cut-off at ∼1.5 Hz (approximating a DC-driven loudness estimator). Each simulation returned the difference between the AM content of the two traces over 1000 trials, and the average of 100 simulations is plotted in Fig 5E (orange shading; see [38] for related results). S5 Fig illustrates this procedure for 1 simulation (see caption for details of individual panels).

We also wished to verify that the slight amplitude deviations introduced by the randomly generated carrier (which was refreshed from block to block, see above) did not affect our calculations based on the notional AM perturbations specified before application of the carrier to generate the stimulus waveform. To this end, we applied the following energy-based recalibration to the stimulus samples in our dataset: each waveform as it was delivered to the listener was split into 9 segments, and RMS (root-mean-square) was computed from each segment to obtain a proxy AM vector equivalent to the 9-element vector specified by our amplitude modulation protocol. We then applied the same kernel estimation procedures used with the pre-specified noise samples to these RMS-corrected samples. The resulting kernel estimates are plotted in S6 Fig, where it can be seen that they demonstrate the same characteristics as obtained before RMS recalibration.

## Results

### Cascade approximation of auditory modulation filter models

Data from reverse correlation experiments are almost invariably interpreted with relation to a cascade that only incorporates one linear filter **L**_1_ applied via template-matching, followed by a threshold conversion to binary decision [49]. However, as discussed in Materials and Methods, this linear-nonlinear (LN) class of models is inadequate for our dataset (we return to this point in Discussion). Instead, we adopt the more general and highly successful linear-nonlinear-linear (**L**_1_**NL**_2_) cascade [64] which, in its most general formulation, serves as a functional approximator of wide applicability [75, 76] (notice that the **L**_1_ linear stage in the **L**_1_**NL**_2_ cascade involves convolution, not template matching; see Materials and Methods for further details). Qualitative inspection of the second-order kernels associated with the experiments described here appears consistent with this class of models [51, 64] (as discussed in Materials and Methods and demonstrated in S2C and S2D Fig).

Both LED and MFB can be cast in the form of **L**_1_**NL**_2_ cascades as illustrated in Fig 2. The only difference between the two models lies in the characteristics of the **L**_1_ stage (blue in Fig 2): low-pass for LED [6] (Fig 2A), primarily band-pass for MFB [16] (Fig 2B). Subsequent stages depend on relatively arbitrary choices of read-out rules [21], but in general they can all be approximated by the combination of a static nonlinearity (**N**) and a subsequent linear stage (**L**_2_). Established techniques in nonlinear system identification [59, 64], combined with idiosyncratic features of their psychophysical variants [51], can be exploited to derive estimates for both **L**_1_ and **L**_2_ from first-order and second-order psychophysical kernels like those shown in Figs 3 and S2 (see Materials and Methods for a more detailed description of the connection between model components **L**_1_/**L**_2_ and psychophysical kernels).

### The band-pass nature of AM filtering

Within the framework outlined above, **L**_1_ and **L**_2_ can be thought of as ‘front-end’ and ‘read-out’ filters. **L**_1_ is the component of primary interest for this study, as it supports the distinction between LED and MFB as being associated with low-pass versus band-pass characteristics respectively (see next section for discussion of recent variants of the MFB model incorporating a lowpass filter [35]). Estimates of this filter are depicted in Fig 3A and 3B (the black trace in A is an estimate of the filter autocorrelation, see Materials and Methods). They show that **L**_1_ presents band-pass characteristics with Q∼1 centred around 8 Hz (thus favouring the MFB model overall). This band-pass property is not an artefactual distortion induced by the target signal, because it is preserved when estimates are obtained from target-absent noise modulations alone (inset to Fig 3B). As we demonstrate in later sections of this study, it is also an evolving characteristic that may not be present at all stages of stimulus exposure.

We quantify this band-pass finding using four different metrics (Fig 4; see Materials and Methods). In *all* cases, **L**_1_ estimate distributions (solid histograms in Fig 4B–4D) fall within the highpass/band-pass range, while **L**_2_ estimate distributions (open histograms) fall within the low-pass range (the latter result points to a late temporal integration window of 50–100 ms consistent with independent estimates from previous studies [21]). More specifically, spectral centroids (Fig 4A) for **L**_1_ are larger than for **L**_2_ (p<10^−5^, unpaired two-tailed Wilcoxon test); Q values (Fig 4B) for **L**_1_ (but not **L**_2_) are significantly larger (p<10^−5^, two-tailed Wilcoxon test) than expected for a uniform spectrum (indicated by dashed lines in Fig 4B); the AC/DC energy ratio (Fig 4C) is larger than 0 (p<10^−5^) for **L**_1_ (indicative of band-pass/highpass characteristics) and smaller than 0 for **L**_2_ (p<0.05); the spectral slope (Fig 4D) is positive for **L**_1_ (p<10^−4^) but negative for **L**_2_ (p<0.01).

It is also noteworthy that results were comparable between datasets from increment detection and decrement detection (y axis): there was no statistically significant difference (at p>0.05) for any metric and for either **L**_1_ or **L**_2_ (data points scatter around solid unity lines in S3 Fig). Such convergence of independent datasets indirectly validates our estimation procedure and suggests that increments and decrements may be processed by the same perceptual mechanism (as also indicated by the similarity in performance metrics, see S1 Fig).

The overall conclusion from the above analyses is that the **L**_1_ filter is band-pass; therefore, AM processing resembles the characteristics of the MFB more than the LED model. This conclusion is not the product of fitting either model to the data: it is based on non-parametric characterization of the front-end filter associated with a general framework cascade that encompasses both models (Fig 2).

### Exposure-mediated retuning of AM filters

All estimates described above were obtained by pooling trials across the entire data collection period undertaken by each listener, spanning several sessions on different days. The characteristics of the perceptual process may have undergone substantial modifications over this extended period, particularly considering that listeners received trial-by-trial feedback and were therefore encouraged to optimize their strategy. To investigate this possibility we defined *early* versus *late* epochs for data collection (see Materials and Methods for definition). The **L**_1_ estimates associated with the two epochs differed: only the ‘late’ estimate (black in Fig 5A) exhibited band-pass characteristics. In contrast, the ‘early’ estimate (green in Fig 5A) was closer to low-pass (see also AM frequency plots within inset). Similar exposure-mediated changes in kernel structure have been previously reported in the vision literature [31, 32]; Fig 5 offers the first demonstration for auditory processing.

The above result is supported by metric analysis of individual listener data: Q estimates (x axis in Fig 5B) are significantly larger (at p<0.01) than the lowpass/highpass cut-off point (orange vertical dashed line) for the late epoch (black), but not for the early epoch (green). We further probed this result with paired data analysis by computing a composite shift index for band-pass characteristics from early to late in each condition and each listener (see Materials and Methods); the resulting distribution (orange in Fig 5D) was significantly shifted away from 0 (p<10^−4^) in the direction of greater band-pass for the late epoch. This shift in band-pass value was accompanied by a significant shift in filter centroid (Fig 5C).

Although more elaborate interpretations are possible, a parsimonious view of our measurements suggests that the shift involved an adjustment of the same underlying filter population, rather than ad-hoc neural assembly of a new filter bank: the ‘late’ dataset overlaps with the ‘early’ dataset in Q-centroid space, only restricted to a smaller region (compare black and green ovals in Fig 5B). When we compare the Q/centroid ranges spanned by the two epochs, we find that the lower percentile boundary (5%) shifts from 0.27 (early) to 0.44 (late) for Q and 2.7 to 5.1 for centroid, but the higher percentile boundary (95%) remains virtually unchanged at 1.5 (Q) and 10 Hz (centroid). A different but equivalent way of conceptualizing this result is to describe the early-late shift as reflecting differential weighting of two discrimination strategies: one relying on loudness, the other on the spectral shape of the modulation frequencies (temporal profile), both driven by valid cues for performing the task (see below for further discussion of this point). In the early phase, the two strategies would coexist and support discrimination to a roughly equal extent; in the late phase, the temporal profile strategy would play a more prominent role.

The above interpretation is consistent with an additional analysis where we estimated coupling between listeners’ choices and the differential DC content (proxy for loudness) of the noise samples presented on those same trials (see Materials and Methods). In the early phase, we found that correlation values across listeners were significantly different than 0 (data points in S4B Fig fall to the right of the vertical dashed line at p<0.02), indicating that the behavioural choices made by listeners were at least partly driven by stimulus loudness. In the late phase, correlation values did not demonstrate a significant shift away from 0 (data points in S4B Fig scatter around the horizontal dashed line at p<0.23), indicating that loudness did not play a significant role in driving behaviour during later phases of data collection.

The filter bank proposed by recent versions of the MFB model [35], encompassing a lowpass filter in the very low modulation range and bandpass filters at higher modulation rates, could accommodate our results when combined with appropriate weighting profiles. In this sense, our data provide support for a mixed lowpass/bandpass version of the MFB model combined with a flexible read-out stage that may undergo internally driven retuning. The lowpass filter recovered by our protocols should not be confounded with the processing stage preceding the filterbank in early formulations of the MFB model [39]; this stage does consist of a lowpass filter, but with a much higher cut-off frequency of 150 Hz. The lowpass filter of interest for the present discussion is therefore best viewed as a subcomponent of the filter-bank itself operating in the very low frequency range, rather than a separate earlier stage extending to the high frequency range.

To gain better insight into the temporal evolution of the exposure-mediated effects, we obtained centroid estimates across 10 different epochs of data collection. Centroid estimates drifted exponentially towards higher values (Fig 5E) matching closely the estimated modulation content of the stimulus (indicated by orange shading, see Materials and Methods and S5 Fig), and this effect was surprisingly robust across listeners: even though drift (see Materials and Methods for definition) returned a noisy measurement for individual listeners (see 95% confidence intervals in Fig 5F), it was consistently positive (symbols fall to the right of vertical line in Fig 5F) so that the overall trend across listeners was highly significant (p<0.005).

The above-detailed modifications of filter structure were associated with only mild improvements of absolute efficiency in some listeners (S4A Fig). This apparent decoupling between filter estimates and performance metrics is a well-documented finding in relation to various perceptual phenomena [77–79] including learning [33]. Direct coupling is theoretically expected only for LN models [80] which are not applicable to our experiments as pointed out earlier; therefore, the estimated filters cannot be transparently linked to discrimination performance. Even if they were, there are at least two reasons why one may not expect to see performance differences.

First, learning effects on AM discrimination are small and difficult to expose (often requiring >100 listeners, see [81]). Second, successful discrimination in our task was supported by both lowpass and bandpass stimulus power (see two peaks in S5I Fig); in this respect our protocol differs from the equally valid ones adopted by previous studies (e.g. [82]) with the specific goal of excluding loudness cues (see Materials and Methods for clarifications as to why we deliberately avoided a stimulus design that would invalidate loudness cues). Because filter structure shifted between these two equivalent sources of task-related information (see above), discrimination performance may well remain unchanged even though supported by different regions of AM frequency.

Prompted by the above results, we re-analyzed data from a prior published study [58] to determine whether similar effects could be exposed for an independent dataset collected using substantially different stimulus/task designs. At the time when this dataset was published, the exposure-mediated effects reported in the present study were not known. We converted perceptual filters from the previous study into a format comparable with the one adopted here and applied the same analysis; as demonstrated in S7 Fig, we obtained remarkably similar results, including lowpass-to-bandpass retuning during the first ∼4K trials.

## Discussion

This study represents the first targeted application of psychophysical reverse correlation to AM processing. Although related tools have been applied successfully in auditory neuroscience [83] and psychoacoustics (see [84] and [85] for the case of spectral processing and [48] for an application to a loudness illusion), they have not addressed the specific case of modulation perception [86], possibly due to multiple challenges associated with this question. First, there is the critical issue of which stimulus dimension should be perturbed by the noisy process in order to provide meaningful and feasible leverage for tapping into the mechanisms responsible for analyzing AM signals. Adding acoustic white-noise is inappropriate, partly because the envelope fluctuations it induces are difficult to control and exercise adequately, and partly because the dimensionality of the space needing characterization is impractically large to measure [86]. Second, there is the question of whether the analytical toolkit associated with reverse correlation is sufficiently flexible to accommodate both LED and MFB: in the vast majority of its applications, reverse correlation is tightly coupled with the assumption of the linear-nonlinear (LN) cascade [49, 87]. Neither LED nor MFB can be correctly approximated by this model (and our data fail to comply with its basic prediction that target-present first-order kernels must match corresponding target-absent estimates, compare black versus orange traces in Fig 3A), requiring more elaborate analytical tools.

To overcome these challenges, we exploited techniques from nonlinear system identification analysis [59] where the coupling between input noise and output response is used not only to compute linear descriptors of the sensory process [55, 85, 87], but also nonlinear (second-order) descriptors that afford the opportunity to characterize more complex cascades than LN [64]. In particular, these additional tools can effectively constrain linear-nonlinear-linear (LNL) cascades [51] to which both LED and MFB likely conform when formulated with reference to the stimulus dimension perturbed in the experiments described here (Fig 2). By combining these system identification tools with AM-tailored stimulus perturbations, we gained sufficient insight into the perceptual process to constrain its properties via data-driven characterization. To appreciate the significance of the analysis adopted here, it is instructive to consider first-order filter estimates from target-absent noise modulations alone (orange trace in Fig 3A). This measurement is similar to the decision weight profiles reported by loudness studies [44–48], including evidence for a primacy effect (larger weights during early phase of the stimulus [47], see orange trace in Fig 3A), and is often regarded as a more appropriate description of the filtering process [88, 89]; more importantly, these measurements (from previous studies as well as our own) present *lowpass* characteristics, with no evidence of bandpass AM filtering. Signatures of bandpass processing are exposed specifically by second-order kernels (Fig 3B). Indeed, when the dataset from [48] is re-analyzed using the nonlinear tools described here, bandpass filtering becomes evident at the level of second-order kernels in the presence of decisively lowpass first-order kernels [90] (see [91, 92] for related examples from the vision literature).

A further enabling factor in the experiments reported here is the stability of task rules and impenetrability of cognitive introspection. Specifically, listeners in these experiments carried out the same task throughout data collection and it is extremely unlikely that from one trial to the next they could explicitly monitor all small deviations introduced by the noisy process and introspect cognitively on those to reach a decision. They therefore operated under relatively stable conditions (except for potential intrinsic changes in adaptive state), allowing us to confidently treat our dataset as reflecting the properties of the same perceptual machinery throughout [31, 32] (even though specific parameters within that machinery may change with exposure). These advantages carry the cost of unusually large data mass (for this study we collected ∼100k trials), restricting our investigation to only a limited ecologically relevant portion (3–12 Hz) of the system’s operating regime, however they allow us to exclude stimulus-driven alterations. More specifically, previous literature has shown that auditory perceptual templates depend not only on signal spectrotemporal structure [26] but also on signal intensity [27] (see [92–94] for related results in the vision literature). Furthermore, strategy shifts not dissimilar from those we report here can be triggered by simple stimulus modifications such as signal-masker asynchrony [28]. It is therefore critical to use stable task rules and a statistically invariant stimulus throughout (as we have done in this study) if one is to ascribe spontaneous filter changes to the perceptual system alone.

Over the past decades, evidence in favour of LED/MFB models [7, 20] has been interpreted in the light of fitting procedures around specified computational implementations and often requiring ad-hoc adjustments for different datasets, weakening the associated conclusions regarding the applicability of one model over the other. Our approach did not favour any specific model, nor did it involve explicit implementation of those or other computational schemes. At the same time, it enabled model selection while retaining close proximity with data structure. These methods uncovered a more complex picture than initially suggested by the LED/MFB dichotomy. Although the low-pass/band-pass distinction retains its descriptive power in relation to our dataset, the underlying mechanism displays *dynamic* adaptive properties that potentially depend on various factors, most notably learning-mediated plasticity [95–99] (see also expectation effects on AM processing [29]). This raises the possibility that previous diverse interpretations of the results (with respect to lowpass versus bandpass filtering) may in fact both provide adequate representations of the underlying process, albeit under different learning states [18]. For example, long-term release from adaptation of postulated bandpass filters [18] may need to be re-interpreted in the context of exposure-mediated reweighting across the filter bank, rather than evidence against low-level adaptation within the MFB (as originally hypothesized in [18]). This interpretation may be verified/falsified using the tools developed and validated in this study.

A further unresolved issue concerning the LED/MFB distinction pertains to the specific Q value associated with AM filtering. It was originally hypothesized that the filterbank associated with the MFB exhibits slightly different Q’s for low versus high modulation rates [16]: above ∼10 Hz, filters would span a bandwidth that increased with center frequency; below ∼10 Hz, bandwidth would remain roughly constant regardless of center frequency. Our dataset presents sufficient variability of estimated central spectroid to span the 3–10 Hz range (y axis in Fig 5B), allowing us to test the latter hypothesis directly. We find that the hypothesized trend is well supported by data: if filter bandwidth is relatively independent of center frequency, the ratio between center frequency and bandwidth (Q) should scale with center frequency; consistent with this prediction, we measured a strong correlation between spectral centroid and Q for both early (r = 0.89,p<10^−7^) and late (r = 0.9,p<10^−6^) epochs (see tilted ovals in Fig 5B). However, the specific values hypothesized for bandwidth (Q≈2; [16]) are higher than suggested by relevant studies (Q≈1; [24, 25]) and by some of the values we measured in this study (Fig 4B). Our results indicate that, even when restricted to comparable regions of AM rates, aggregate Q values may span a 0.3–1.7 range depending on the degree of recalibration undergone by the system via exposure/learning (see data scatter across x axis in Fig 5B), consistent with the latest estimates [24, 25] but substantially lower than the values employed by recent modelling work [35, 39].

To what extent do our results depend on the stimulus parameters and task specifications selected for this study? As explained earlier, we were constrained in our ability to test a wide range of configurations, however we did perform measurements using increment as well as decrement target signals. The spectral profile of task-relevant stimulus information is substantially different between these two configurations: in the increment case, the useful bandpass region lies between 8 and 20 Hz (S5I Fig), while the decrement configuration mostly targets the 4–8 Hz region (S5J Fig). As for the lowpass (loudness) cue, it required opposite read-out rules for the two configurations (target signal is louder in the increment configuration, and softer in the decrement configuration; see peaks of opposite signs in the lowpass region of S5I and S5J Fig). Despite these differences in the stimulus, we observed no difference between increment and decrement estimates of bandpass tuning (S2 and S3 Figs), and we found no correlation/relationship between the exposure-mediated effects (Fig 5E and 5F) and the relative exposure to increment versus decrement signals. Furthermore, the very fact that these characteristics (both centroid and Q values, Fig 5B–5D) changed with exposure in the face of an unchanging stimulus indicates that they are not solely driven by stimulus specification. This is not to say that our estimates are completely decoupled from the chosen stimulus/task parameters: by requiring observers to detect a specific signal, we are implicitly prompting them to calibrate their available perceptual resources in relation to the assigned task and signal [92, 94]; for example it is conceivable that, in extreme cases such as detection of very narrow AM pulses with a broad modulation spectrum, the measured filters may become broader reflecting stimulus characteristics [93]. However, beyond the inevitable structure imposed by task instructions and signal specification on listeners’ selection of perceptual resources, our measurements appear to reflect properties that are instrinsic to the perceptual process and informative of its inherent characteristics. Finally, targeted re-analysis of an earlier published dataset [58] exposed structure entirely consistent with the results reported here (S7 Fig), providing strong validation of our findings: due to numerous design differences between the two studies, it is not trivially expected that we should find similar overall characteristics.

In summary, the experimental approach adopted in this study has enabled us to examine outstanding issues in the AM processing literature from a different perspective, and clarify important aspects of this phenomenon, enabled by a set of tools that has not been previously applied with relation to this phenomenon. We have delineated the relationship between LED and MFB models within the context of a prominent theoretical cascade framework [100], we have refined and further constrained previous estimates of channel selectivity for processing amplitude modulations, and we have demonstrated its spontaneous adaptive nature in the context of active listening tasks (see also [31, 32]). Further research and additional characterization will be necessary to establish the applicability of our findings across a wider range of tasks and determine the exact nature and functional purpose of exposure-dependent adaptive processes [101, 102].

## Supporting Information

S1 Fig Performance metrics are similar for detecting increments and decrements. (A) Sensitivity is in the d′∼1 range (unity is indicated by dashed lines). (B) Bias is not statistically different from 0 (indicated by dashed lines). (C) Internal noise is within expected range [56] (grey shaded area). (D) Absolute efficiency is within expected range. Ovals are aligned with best-fit line, with radii matched to 0.5× (thickest), 1× and 1.5× (thinnest) SD’s for data projected parallel and orthogonal to line. Different symbol shapes refer to different listeners. Error bars plot ±1 SEM. There was no statistically significant difference between increment (x axis) and decrement data (y axis) for any of these metrics.
(EPS)

S2 Fig Detailed overview of kernel measurements. (A,B) Target-absent and target-present first-order kernels are plotted in A and B respectively (see Materials and Methods for detailed description of how these kernels were computed), for both increment (black) and decrement (red) detection tasks (shaded regions show ±1 SEM). Target-absent kernels differ markedly from target-present kernels (among other features, notice different scaling of y axis between A and B), ruling out the linear-nonlinear model typically assumed by reverse correlation studies. Inset in (B) shows the autocorrelation of the kernels in E. (C,D) Second-order kernels for increment and decrement detection (plotted as upper/lower triangular matrix respectively). (E) First row (or equivalently column) from second-order kernels (indicated by black/red rectangles in C-D). Inset shows same measurements but only from target-absent second-order kernels.
(EPS)

S3 Fig Expanded view of bandpass characteristics for front-end filter (L_1_) and read-out filter (L_2_). Panels (A-D) display estimates from increment detection on x axis against those from decrement detection on y axis. Solid symbols refer to **L**_1_ (bigger symbols for estimates obtained from first row/column of second-order kernels (S2E Fig) and smaller symbols for estimates obtained from target-present first-order kernels (S2B Fig)). Open symbols refer to **L**_2_. Different symbol shapes refer to different listeners. Error bars plot ±1 SEM. As for performance metrics (S1 Fig), there was no statistically significant difference between increment (x axis) and decrement data (y axis) for any of these estimates.
(EPS)

S4 Fig (A) Absolute efficiency [61] was similar during early (x axis) and late phases. (B) Coupling between noise-driven DC fluctuations and behavioural responses, however, was only present during the early phase (see Materials and Methods and main text).
(EPS)

S5 Fig Bandwidth characteristics of signal and noise. The stimulus waveform (A) is mapped to its spectrotemporal representation (B) using standard cochlear parameters [103]. It is then processed by a bank of modulation 1-octave filters with central frequency ranging from lowpass to 64 Hz (C); the scalar output of each filter (plotted in D) is the mean/maximum (orange/black) absolute value across the corresponding spectrogram (mean trace has been rescaled by 3× to span range comparable to maximum trace). This procedure is applied to both signal+noise (A-D) and noise-only (E-H) stimuli (example shown for increment detection in A-I); the difference between the filter-bank output from the two stimuli provides an indication of the modulation frequency bands that supported above-chance discrimination (the region around lowpass (lp) and 8–16 Hz for increment in I, and 4–8 Hz for decrement in J). Solid lines in D,H-J show mean across 20 iterations of 5K trials each (±10 SD indicated by shaded region).
(EPS)

S6 Fig Fine temporal structure does not impact our results. As detailed in Materials and Methods, a new carrier was generated at the beginning of each blok of data collection. This additional source of randomness in the stimulus caused slight amplitude deviations from those specified by the AM noise deliberately introduced and controlled by the experimenter. To verify that these deviations had no impact on our results, we reconstructed the amplitude content of individual noise samples while taking into account the specific carrier waveform presented on those specific trials (see Materials and Methods). In this figure, we reproduce the critical measurements supporting our conclusions (target-present first-order kernels (left column) and **L**_1_ estimates from second-order kernels) before and after correcting for carrier-induced AM deviations (black/red versus blue/yellow respectively). More specifically, A-B plot the quantities estimated in Fig 3A and 3B for increment detection; C-D plot the same for decrement detection. Shaded regions show ±1 SEM. The overlap between corrected and uncorrected estimates in A is such that the two traces (blue versus black) are barely distinguishable in the plot.
(EPS)

S7 Fig Confirmation of lowpass-to-bandpass shift from post-hoc analysis of prior dataset. We re-analyzed data from a previously published study [58] in light of the results obtained here. More specifically, we converted spectrograms from reference [58] (defined across the dimensions of both time and frequency) into vectors defined along time alone by extracting values only at the target frequency, to bring them into coarse alignment with the perceptual filters used in the present study. Except for small differences in total duration and sampling rate (7 samples spanning 280 ms in the prior study versus 9 samples spanning 300 ms in the present study), results from the two studies are therefore comparable in data format (even though different stimuli (in particular target signals) and tasks were employed for the two studies). A is plotted to the same conventions adopted in Fig 3A. B is plotted to the conventions adopted in the inset to Fig 5A. To make the early/late analysis comparable to the analysis used in Fig 5, we defined the ‘early’ phase as spanning the first 2000 trials (from trial 1 to 2000) and the ‘late’ phase as spanning the second 2000 trials (from trial 2001 to 4000) for each of the 8 observers tested in the prior study (main two-tone condition). This analysis exposes structure in the previously published dataset consistent with the effects reported in the present study, further supporting their validity and applicability to other stimuli/tasks.
(EPS)
